# 3D printing and milling a real-time PCR device for infectious disease diagnostics

**DOI:** 10.1371/journal.pone.0179133

**Published:** 2017-06-06

**Authors:** Geoffrey Mulberry, Kevin A. White, Manjusha Vaidya, Kiminobu Sugaya, Brian N. Kim

**Affiliations:** 1 Department of Electrical & Computer Engineering, College of Engineering and Computer Science, University of Central Florida, Orlando, Florida, United States of America; 2 Burnett School of Biomedical Sciences, College of Medicine, University of Central Florida, Orlando, Florida, United States of America; University of Helsinki, FINLAND

## Abstract

Diagnosing infectious diseases using quantitative polymerase chain reaction (qPCR) offers a conclusive result in determining the infection, the strain or type of pathogen, and the level of infection. However, due to the high-cost instrumentation involved and the complexity in maintenance, it is rarely used in the field to make a quick turnaround diagnosis. In order to provide a higher level of accessibility than current qPCR devices, a set of 3D manufacturing methods is explored as a possible option to fabricate a low-cost and portable qPCR device. The key advantage of this approach is the ability to upload the digital format of the design files on the internet for wide distribution so that people at any location can simply download and feed into their 3D printers for quick manufacturing. The material and design are carefully selected to minimize the number of custom parts that depend on advanced manufacturing processes which lower accessibility. The presented 3D manufactured qPCR device is tested with 20-μL samples that contain various concentrations of lentivirus, the same type as HIV. A reverse-transcription step is a part of the device’s operation, which takes place prior to the qPCR step to reverse transcribe the target RNA from the lentivirus into complementary DNA (cDNA). This is immediately followed by qPCR which quantifies the target sequence molecules in the sample during the PCR amplification process. The entire process of thermal control and time-coordinated fluorescence reading is automated by closed-loop feedback and a microcontroller. The resulting device is portable and battery-operated, with a size of 12 × 7 × 6 cm^3^ and mass of only 214 g. By uploading and sharing the design files online, the presented low-cost qPCR device may provide easier access to a robust diagnosis protocol for various infectious diseases, such as HIV and malaria.

## Introduction

Early diagnosis and prompt treatment is crucial to provide the best health care to a patient and to reduce the risk of further spreading, especially in cases involving malaria parasites, tuberculosis (TB), and human immunodeficiency virus (HIV). For this reason, many different techniques have been introduced and developed under the category, rapid diagnostics tests (RDT). The RDT offers low manufacturing cost and provides the first-level screening to determine the necessity of follow-up tests. A typical tradeoff from the RDT methods is accuracy and lack of quantification to obtain low-cost and ease of use. For example, the RDT devices for malaria detection, measures specific antigens, such as Histidine Rich Protein II, pLDH, and pAldo, produced from malaria parasites [1]. The antigen-based detection device typically has non-specific bindings that produce false positive results, and yields low accuracy. Also, most antigen-based devices require a relatively high-level of pathogens in the patient sample compared to Polymerase Chain Reaction (PCR) to be detected as a positive result. The World Health Organization (WHO) recommends at least a 75% panel detection score, the detection success rate for low parasite density samples (200 parasites/μL), and a false positive rate of less than 10% [2]. Even for the commercially-available RDT devices that satisfy these criteria, the panel detection score is marginally higher than the recommended score, and false positive results exist. For HIV RDTs, antibodies for HIV-1 and HIV-2 are often measured, and a strategy to combine the detection of HIV-1/2 is recommended. Over the last generations of HIV RDT, the HIV antigen detection is incorporated with the antibody detection in attempt to detect the HIV infection in an early phase [3]. However, it lacks quantitative result and only outputs positive or negative. For acute HIV infection (AHI), an early HIV infection, most antigen- and antibody-based RDT fail to report the infection because the antigen and antibody biomarkers are not present in that phase [3]. The only diagnosis mechanism that can measure the presence of HIV infection is nucleic acid-based and detects the presence of HIV RNA [4].

According to the Centers for Disease Control and Prevention (CDC) and WHO, for diseases such as Malaria and HIV, the use of PCR is highly recommended to obtain results and to determine the proper treatment [4,5], because PCR reveals both the presence and the quantity of the pathogen and have high sensitivity and specificity. However, alternative techniques such as RDT are often used to make a diagnosis because of the more accessible nature of those techniques. The quantitative PCR (qPCR) is a robust technique that amplifies the target DNA sequence, while monitoring the amplitude of signal originating from DNA-intercalating probes, typically fluorescence, to quantify the amount of DNA being amplified [6,7]. With custom-designed primers, PCR can be designed to amplify a specific sequence of DNA, providing high specificity in diagnostics. This is useful in differentiating various strains of malarial infection to coordinate proper treatment. The characteristic of the fluorescence signal reveals the original quantity in the sample before amplification. From the early phase of HIV infection, qPCR is capable of detection and characterization of the low-level infection. Theoretically, a well-designed PCR reaction, with low non-specific binding, can detect the presence of a single DNA molecule in the reaction tube [8]. However, a PCR assay presents a major logistical challenge for primary clinics, requiring equipment such as a clean bench, thermocycler and detection module that can cost several tens of thousands of dollars. Furthermore, the cost increases to maintain such equipment and facilities. PCR involves the heating and cooling of a sample containing target DNA and reagents upwards of 40 times. Because of this cycling process, a complete PCR reaction involves a reasonably long amount of time, usually around 2 hours, a fact which causes most PCR machines to be designed to hold many samples at one time. This greatly increases the size of these machines, which in turn raises the price. Due to such difficulty, many clinicians especially in a tight budget condition frequently skip confirmatory diagnosis on many occasions and prescribe medicine either based on a relatively inaccurate diagnostic method or pursue empirical treatments based on vague symptoms without adequately ruling out differential diagnoses [9]. This practice has several disadvantages such as increasing the chance of wasting a limited supply of medications and increasing antibiotic/antiparasitic drug resistance in a local community. Treating patients empirically or based on comparatively inaccurate diagnostic methods also increases a chance to have a delayed diagnosis, especially in early stages of infection or in the asymptomatic phase. Complications from delayed diagnosis include irreversible organ damage, increased usage of medical supplies to contain exacerbated symptoms and failure to quarantine early. For instance, *P*. *falciparum* can cause ischemic insults to the brain, lungs and kidneys when not treated promptly which frequently can cause mortality to children and irreversible damage even if patients recover from a disease. When patients enter the advanced clinical phase from infectious diseases, hospitalization with intravenous support/patient monitoring is required. In addition, an antibiotic/antiparasitic is frequently required to deal with systemic inflammation caused by an infectious agent. Containment for HIV in a community level is always challenged by frequent quarantine failures due to undiagnosed/misdiagnosed asymptomatic carriers. Having access to low-cost, easy to manufacture and low-maintenance PCR equipment at a primary doctor level enables rapid on-site diagnosis which brings a great benefit to community health. Treatments can be given before infection causes more damage to patients. The clinic can be more efficient on distributing their limited medical resources and authorities can set up quarantine for rapid containment of infectious disease if needed.

Previously, attempts have been made to lower the cost and increase the accessibility of PCR instrumentations [10–14]. A small chip with a heater and temperature sensor was fabricated by silicon micromachining, and was integrated into a handheld device to perform qPCR in under six minutes [11,15]. A pocket-sized PCR thermocycler was designed by fabricating heating blocks with various temperature for convective-flow of PCR samples [13]. A real-time PCR device was made using a micro-fabricated glass device and 3D printer [14]. Most devices developed under this motivation had custom parts that needed to be manufactured with unique or expensive processes, such as printed circuit board (PCB), microfluidics, and micro-fabricated devices, in order to miniaturize the size of the device for portability (accessibility). Recently, 3D printing technology has been adapted to the medical device manufacturing [14,16,17]. The main reason is to reduce the cost of manufacturing medical devices and to increase the accessibility in various places around the globe through a simple process of design sharing over the internet. Microfluidic devices were fabricated using inkjet 3D printing methods as well as stereolithography [16]. A custom-built microfluidic, suitable for cultivating Methicillin-resistant Staphylococcus aureus (MRSA), was used to detect the presence of MRSA with a gold nanoparticle-based assay [17,18].

**Fig 1:**
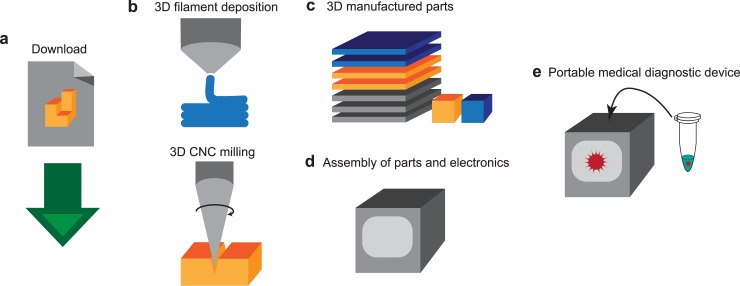
From digital design file to portable diagnostics device. (a) A digital format of design file is uploaded to the internet, and downloaded by users. (b) A user uploads the digital files into 3D printer and 3D CNC milling machine for manufacturing. The 3D printer extrudes and deposits filament and the 3D CNC mill cuts and drills through material to engrave patterns and shapes. (c) This results in 3D manufactured parts for assembly. (d) The parts and off-the-shelf electronics components are assembled. (e) This assembled device can function as a portable medical diagnostic device for detecting and quantifying the pathogen in a sample through qPCR.

The goal of this research is to design a low-cost 3D manufacturing method to fabricate a portable qPCR device (Fig 1). Thus, the digital format of design files can be shared over the internet, which enables any person to easily download the files and produce a qPCR device (Fig 1A). The open-source instrumentation allows wide distribution of valuable methods [19–22], in this case, a medical diagnostics device. For the manufacturing (Fig 1B), a simple 3D filament deposition printer and 3D CNC milling device are selected, because of their wide availability and low-cost (ranging from $300 - $4,000). For example: the Wanhao Duplicator I3 3D printer is priced at $379, the Inventables X-Carve CNC mill is priced between $1328 and $1608, and the ZMorph 2.0SX 3D printer / 3D CNC mill used to manufacture this device is priced between $2690 and $3440. Additionally, if the person downloading the files is unable to access these 3D manufacturing tools, they can elicit the help of a third party. The device, resulting from 3D manufacturing, can perform real-time PCR for amplifying while monitoring the target nucleic acid sequence, and conclusively detect as well as quantify an infection of pathogens (Fig 1E). This study grants easy access to high-quality biochemical instrumentation on resource-limited settings for medical diagnostics.

## Materials and methods

The qPCR printed device consists of custom designed parts, such as a heating element, circuit board, and fixtures for the light path and casing. All of the custom parts are designed for ease of manufacturability and no dependency on advanced manufacturing infrastructure or equipment, most of which are 3D printed. The tools used for custom manufacturing are a 3D printer, a 3D milling machine, and a hand-drill. The casing for the overall device, the holder for the heating element, the fan and cooling system as well as the structure of the light path for fluorescence reading are made of 3D fused-filament deposition parts. A 3D CNC mill is used to fabricate the circuit board for the electronic controls. The device is designed to use a typical PCR tube that has several key features, including a complete seal for inhibiting evaporation of the sample and high optical transparency for the excitation and emission wavelengths. The details of the design and manufacturing of custom parts are described in the following section. The mechanical parts of the device are designed using AutoDesk Inventor, a 3D computer-aided design (CAD) software. Using Inventor enabled the device to be designed as compact as possible by testing multiple designs without having to manufacture them. When a part is ready to be made, it is simply printed using the 3D printer or milled using the 3D CNC mill.

### Heating element design

**Fig 2:**
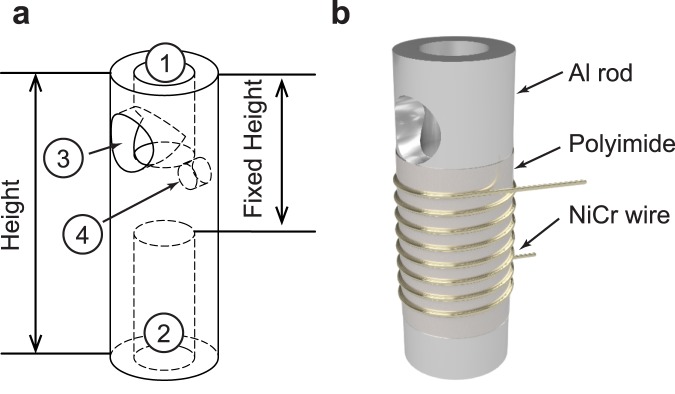
The aluminum heating element for qPCR device. (a) The aluminum rod has four holes for ① PCR tube insertion, ② convection cooling, ③ the excitation light path, and ④ thermistor insertion. (b) The heating element consists of an aluminum rod, a layer of polyimide film, and a coil of NiCr wire. The dimension and location of the holes are: ① ⌀ is 3.72 mm and depth is 5.46 mm, ② ⌀ is 3.72 mm and depth is 8.59 mm, ③ ⌀ is 3.56 mm and location from top is 3.70 mm, and ④ ⌀ is 1.59 mm and depth is 1.00 mm and location from top is 3.70 mm.

The heating element is based on a section of aluminum rod with holes axially bored in from the top and bottom (Fig 2A). One of these holes (① in Fig 2A) allows for the insertion of the PCR tube and is a depth and diameter which allows the portion of the PCR tube containing the reaction solution to be fully inserted. The other (② in Fig 2A) is used to increase the surface area of the rod while simultaneously decreasing the rod’s volume. This is done so that both heating and cooling times are minimized. On the sides of the rod there are two additional holes. One (③ in Fig 2A) is located where the PCR tube is inserted and allows for light to excite the sample. The other (④ in Fig 2A) is located in the middle of the rod and houses a thermistor used to measure the temperature of the heating element. This aluminum rod is covered with polyimide tape that provides electrical insulation and is then wrapped with 7 turns of 0.255 mm diameter NiCr wire. This number of turns was chosen to result in a length of wire with a resistance of ~5.6 Ω that will draw ~1.3 A from the 7.4 V battery, which is below the maximum current draw of 1.6 A that can be safely used with the chosen lithium-ion batteries as per the manufacturer’s datasheet. The current passed through the NiCr wire dissipates ~11.8 W and thus releases heat which is used to raise the temperature of the aluminum rod, a process known as Joule heating. The resulting heating system has a time constant of ~9.133s.

To cool the heating element, a centrifugal fan is employed. The fan consists of a small brushed DC motor that is controlled by the microcontroller using a relay. Since electrical noise is introduced onto a portable power supply when a DC motor is operating, the motor is connected to a network of capacitors. Without these capacitors, the noise created by the motor dramatically degrades the readings from sensors using the same power source, in this case, the photodiode and thermistor. The motor drives a small 3D-printed impeller, which is the main element of the 3D-printed centrifugal fan. This impeller pulls in air at ambient temperature from the bottom of the device and accelerates it towards the aluminum heating block located in the cartridge. This process aids in rapidly lowering the temperature of the heating block, and thus, the sample. The more efficient cooling process using the fan allows for the reduction in time it takes to complete a PCR amplification. The resulting cooling system has a time constant of ~20.97s. The dimensions of the heating element are optimized using thermal simulations.

Thermal simulation of the heating elementTo achieve easy manufacturability of the heating element, the section of aluminum forming the heating block is cut from a 6.35 mm diameter aluminum rod using a saw, allowing for a variable height and a fixed outer diameter. In order to determine the optimal height of the heating element, thermal simulations are performed in COMSOL Multiphysics 5.2a to determine the effects of the element’s height on the duration of heating and cooling cycles. Increasing the height of the heating element, whilst maintaining the hole diameters and distance between the top (① in Fig 2A) and bottom holes (② in Fig 2A), will increase the surface area of the rod, thus improving convection and reducing the cooling period. Additionally, the increase in height will increase the volume of the aluminum, thus increasing the heat capacity and the heating period. The simulation model consists of an aluminum rod and polyimide tape. The Heat Transfer in Solids interface in COMSOL Multiphysics is used to study the thermal conduction and convection. In the heating simulations, the joule heating from the NiCr wire is applied to the polyimide tape as thermal power. The thermal power applied is determined by measuring the power dissipated by the NiCr wire during heating cycles. In the heating simulations, a fan-less operation is assumed, while the cooling simulations are performed with a higher convection coefficient to model a fan. The convection coefficients and polyimide tape thickness, which accounts for air pockets between layers of tape in the experimental setup, are defined so that the simulation cycle time (heating and cooling) matches the cycle time measured in our preliminary data. The determined convection coefficient and polyimide tape thickness for the heating stimulations are 15.8 W/(m^2^·K) and 0.73 mm, respectively. For the cooling simulations, the convection coefficient is 69.1 W/(m^2^·K) and the polyimide tape thickness is the same.

### Optical design of real-time fluorescence measurement

**Fig 3:**
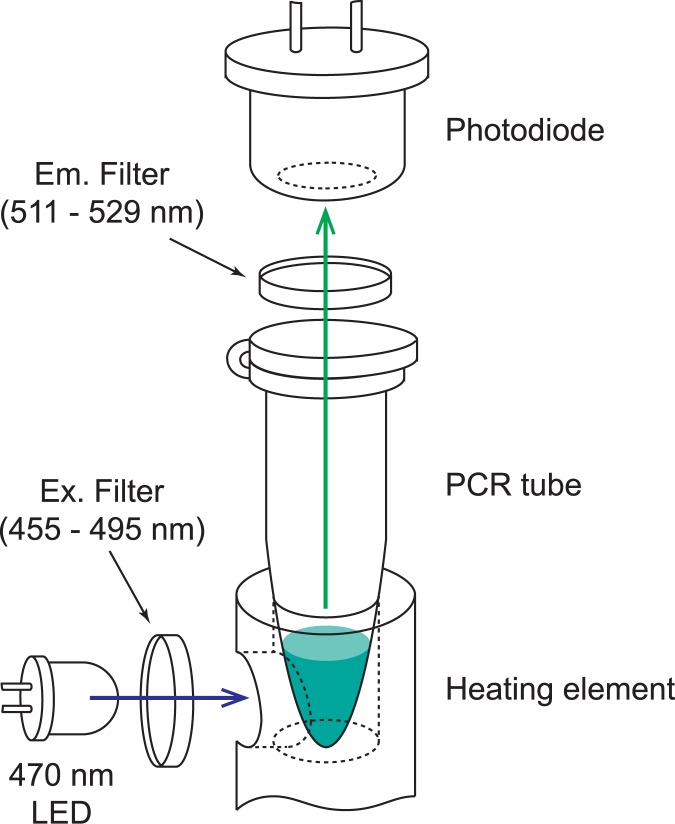
Optical design of real-time fluorescence measurement for qPCR. The blue LED (470 nm) emits excitation wavelength that is filtered through an excitation filter which is a band-pass filter of 455 to 495 nm. The excitation light travels through the hole in the aluminum rod and is absorbed to the sample in the PCR tube. The emitted light is collected by an avalanche photodiode through an emission filter, with a band-pass at 511 to 529 nm.

The optical components are critical to the functionality of a real-time PCR system as they provide the means of determining the concentration of amplified target DNA. The system consists of a photodiode and emission filter located coaxially to the PCR tube, and an LED and excitation filter located adjacent to the sample (Fig 3). All the components used to position these optical parts in the correct locations are 3D printed. The LED emits blue light through the blue excitation filter and travels through a hole in the side of the aluminum heating block and finally illuminates the sample located in the PCR tube. The intercalating dyes in the sample emit green light when exposed to this blue excitation light in an intensity proportional to the concentration of the target DNA. This green fluorescence light travels up through the lid of the PCR tube, through the emission filter, and arrives the photodiode sensor. The photodiode produces a voltage corresponding to the fluorescence intensity emitted by the sample. This voltage is then amplified and read by the microcontroller. The resulting system is able to quantify the amplification of DNA produced by qPCR. This optical design adapts a simple separation of the emission and excitation light paths to reduce the direct injection of excitation wavelength into the photodiode. In a typical qPCR device, this is accomplished by using a dichroic mirror with a significantly separated property of reflection and transmission wavelength. However, avoiding to use a dichroic mirror simplifies the optical path design and reduces cost. Additionally, the optical filters and LED can be chosen to operate with various fluorescent dyes. For the components in this design, detailed in supplemental data S1 Table, the optics are compatible with both FAM probes as well as SYBR green dye.

### 3D printing and 3D milling for easy manufacturability

**Fig 4:**
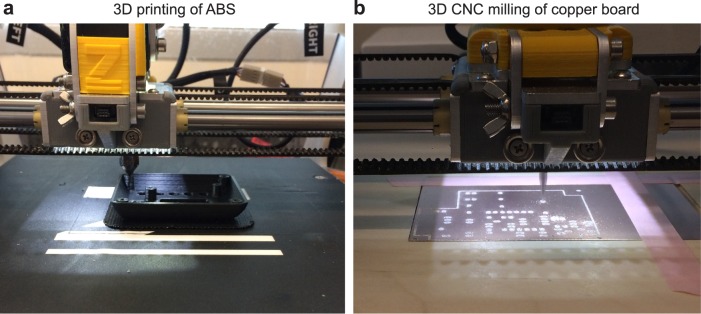
Photographs of 3D printing and 3D milling of all the parts using a multitool 3D printer. (a) The faceplate is being printed with black ABS material on a heated bed. (b) The main board is being engraved using 3D CNC mill.

All the 3D-printed enclosures, holders, and parts are 3D printed using the Zmorph 2.0 SX multitool 3D printer (Fig 4A). For 3D printing, a 1.75 mm black ABS filament is used. This is chosen because of ABS plastic’s relatively high strength compared to PLA, the other common 3D printing plastic. Black filament is used so that the case and optical components are opaque to avoid interference with the fluorescent measurements. The printing is performed with a 0.4 mm nozzle and a 0.2 mm layer height which results in a smooth surface finish, with deviations from the CAD model being less than 0.2 mm. The walls of the main case are 1.6 mm thick resulting in a structure that can support a mass of 70 kg. The Zmorph tool offers the capability to switch out the tool head to carry out different tasks, such as 3D CNC milling and paste extrusion. To integrate the heating, cooling, and optical systems, a circuit board is made to connect all elements together. The circuit board has been made using an isolation milling process with the Zmorph 2.0 SX multitool 3D printer (Fig 4B). This method uses the milling tool on the printer to cut slots and holes in a piece of laminated copper board. The un-milled copper that remains forms the electrical contacts for the circuit. The electrical components are then fixed to the board by hand soldering, a process which can easily be learned by the average person. Utilizing the process of isolation milling avoids the use of another method such as a photolithographic process, which requires more equipment including a dark room for sensitizing boards, transparency printer, a UV light source for board exposure, an acid-based chemical bath for etching, and a drill press for creating holes. While effective, the difficulty of the photochemical method makes it unachievable for many. Isolation milling avoids unnecessary equipment, careful training, and hazardous chemicals. For those who do not have access to a 3D printer or milling machine, access to this low-cost qPCR machine is still a possibility. Many universities and machine shops have these 3D manufacturing machines available and will allow access to them for individual uses. Providing the files to these establishments will allow them to manufacture the parts for nothing more than a small fee. In the case of the PCB, there are numerous board houses that will accept the files and mail back finished boards for a relatively low-cost.

### Electronics and closed-loop feedback

The main part of the electronic control system is the SparkFun MicroView. The MicroView is a microcontroller with a built in OLED Screen. The microcontroller is used to control the two relays that operate the fan motor and the NiCr heating wire, and is also responsible for turning on the blue excitation LED and for obtaining a reading from the photodiode. Additionally, the microcontroller takes in input from the user via a set of buttons and delivers output to the user by way of the built in OLED screen. The screen displays several menu-based interfaces to the user. These include: “New PCR” and “View Data”. “New PCR” begins the thermocycling and displays the current temperature reading, the current cycle number, the most recent fluorescent measurement, and a plot of the temperature in real time. “View Data” shows the fluorescent measurements for each cycle number in both a tabular and graphical format and can be accessed while the PCR reaction is taking place. The software that controls the operation of the device and runs on the microcontroller is responsible for reading the buttons, controlling the heater and fan, enabling the LED, measuring the fluorescent reading, and displaying information on the screen. The temperature control works using a thermistor mounted on the aluminum heating block. This thermistor is connected in series with a 10 kΩ resistor and creates a voltage divider. The result is a voltage that is proportional to the temperature of the aluminum block. This voltage is read by the microcontroller and converted to degrees Celsius. When in the thermocycling mode, the microcontroller enables the heater. Once the temperature reaches a high threshold, the fan is enabled and the heater is disabled. The fan blows ambient temperature air over the aluminum block and cools until the low threshold is reached. The fan is then disabled and the heater enabled for short pulses until the temperature inside of the tube finally reaches the low threshold. At this point, the LED is enabled and 5 readings are taken from the photodiode. The median of these five samples is recorded to avoid any errors caused by noise on the reading. All the electronic components used are listed in S1 Table in supporting information.

### Sample preparation and detection of lentivirus

Lentivirus, a virus family that HIV belongs to, is used to test the 3D manufactured qPCR device. The lentivirus containing a mouse *SOX2* gene sequence [23], which is targeted for amplification and detection. Real-time Reverse-Transcription Polymerase Chain Reaction (RT-qPCR) is performed. Initially, the RNA is reverse transcribed into cDNA, and the qPCR cycle amplifies the amount of DNA and monitors the corresponding fluorescence changes. The purified virus sample (2 μL) of various concentration tested is mixed into a typical PCR tube with RT-PCR reagents and TaqMan Gene Expression Assay, Mm00488369_s1, that targets 68 bp of the mouse *SOX2* gene. The lentivirus, containing the mouse *SOX2* gene, is chosen to reduce risk of HIV infection to the researcher during this work, while still being able to simulate the detection of lentivirus RNA. Three virus concentrations were tested, 2 × 10^7^ vp/mL, 2 × 10^6^ vp/mL, and 2 × 10^5^ vp/mL, to determine the quantification capability of the presented device. These viral loads are clinically relevant for HIV infection [24]. The total volume of a reaction mix is 20 μL. The reverse transcription step is performed at 55°C for 10 minutes, and the PCR cycles are performed at 95°C for denaturing and at 60°C for annealing and extension. The lysis of lentivirus is not conducted separately, and is integrated as a part of reverse transcription step. To confirm the product length of the qPCR, a gel electrophoresis is used. A 3% Agarose gel (50 mL of 1× TBE, 1500 mg Agarose powder and 5 μL of 10,000× SYBR safe gel stain), with a channel length of 100 mm is ran at 75V for approximately 2 hours.

## Results

### 3D assembly of the system

**Fig 5:**
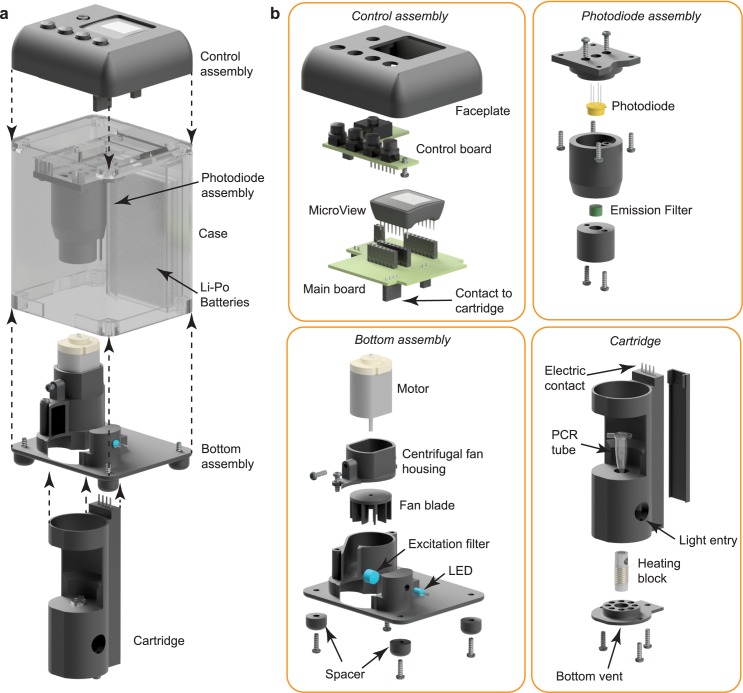
3D assembly of the system with exploded views. (a) The complete device showing locations of Control assembly, Photodiode assembly, Bottom assembly, Case, and Li-Po batteries. (b) Detail of assemblies. The Control assembly contains a faceplate, a control board, a main board, and a MicroView microcontroller. This assembly provides a user interface and houses all electronic controls. The Photodiode assembly contains the photodiode and emission filter. This assembly is used to determine the target DNA concentration present in the sample. The bottom assembly contains a motor, a fan blade, spacers, and a centrifugal fan housing, forming the cooling fan system. It also contains an LED and excitation filter, establishing the light source for illuminating the sample. The cartridge is a removable assembly allowing for easy insertion of the PCR tube containing the sample. It contains the heating block, electrical contacts for connection to the main board, and a vented bottom plate allowing for the escape of warm air.

The structure of the system consists of four assemblies and a case enclosing them. An exploded view of the system is shown in Fig 5A, indicating the placement of all components with respect to each other. The detailed structures of each assembly, control assembly, photodiode assembly, bottom assembly, and cartridge assembly, are presented in Fig 5B. The control assembly contains of all of the electronics that control the device as well as the user interface. This assembly is made of two circuit boards and a faceplate. The lower main board holds the MicroView, photodiode amplifier, and heater and fan relays, as well as the connectors for the cartridge, fan, LED, and upper control board. The control board contains the connection to the battery, the power switch, and control buttons. This assembly is mounted to the top of the case with screws. Under this assembly and mounted to the case is the photodiode assembly. This assembly houses the photodiode as well as the green emission filter. The main housing for the photodiode assembly also contains a tapered portion that helps to align the cartridge with the photodiode when inserted. The bottom assembly houses the fan, the LED and the blue excitation filter. The opening in the bottom cover allows for the insertion and removal of the cartridge. The cartridge consists of a cylindrical shaped housing which accepts a typical PCR tube. On the lower side of the cartridge is a cover with several holes, the central hole holds the aluminum heating block while the outer holes allow for warm air to escape. Along the back side of the cartridge is a channel that holds the wires for the heating element and the thermistor to reach the connector on the top. This connector makes contact with the main circuit board when the cartridge is inserted into the case. Two holes in the side of the cartridge allow for the entrance of air on the left side and for the entrance of blue light from the right. The control, photodiode, and bottom assemblies are mounted to the case. The case also holds two lithium polymer batteries used to power the device. The digital file used for this assembly is shown in S1 Fig and uploaded as S1 File and S4 File in supporting information.

### Optimization of thermocycling

**Fig 6:**
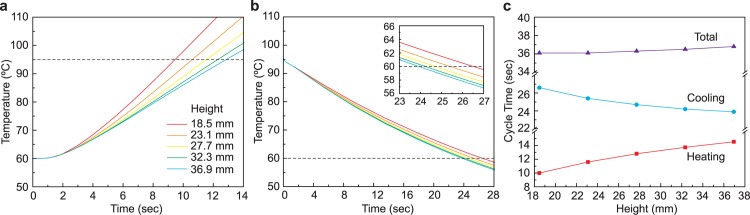
The thermal simulation of the heating element’s heating and cooling cycle time. (a) The heating time from 60°C to 95°C in various heights of heating element. The shortest (9.5 seconds) to heat is 18.5 mm in height. (b) The cooling time from 95°C to 60°C in a fan-operating condition. The shortest (23.9 seconds) to cool is 36.9 mm in height. Increasing the height of the aluminum rod increases the surface area, resulting in improved convection and the length of the cooling period is reduced. The subset graph is an expended view at 23 to 27 seconds. (c) The summary of heating and cooling time for various heights of heating element. The height with the shortest total cycling time is 18.5 mm (36.1 seconds). The heating period increases at a faster rate to the increase in height than the cooling period decreases.

Simulations are performed to optimize the height of the rod used in the heating element to minimize the duration of themocycling. To do this, the height of the rod is varied. The smallest presented height of 18.5 mm (Fig 6) is the practical limitation for the aluminum rod to retain manufacturability with spacing for NiCr wire and holes. The heating periods are 9.5, 10.7, 11.6, 12.3, and 12.9 seconds for the heights 18.5, 23.1, 27.7, 32.3, and 36.9 mm respectively (Fig 6A and 6C). The increase in the heating period is directly related to the aluminum rod height as predicted. For the heights 18.5, 23.1, 27.7, 32.3, and 36.9 mm the cooling periods are 26.6, 25.4, 24.7, 24.2, and 23.9 seconds respectively (Fig 6B and 6C). As expected, the decrease in the cooling period is inversely related to the aluminum rod height. Although the heating and cooling periods react predictably to changes in height, the rate at which the cooling period decreases is lesser than the rate at which the heating period increases. This provides a motivation to decrease, rather than increase, the height of the heating element to reduce the period of a thermocycle. For a fanless system the contrary is true and there is motivation to increase the height of the heating element to decrease the period of a thermocycle. To achieve the shortest thermocycle period, the optimal height of the aluminum rod is 18.5 mm.

### Feedback calibration of thermocycling

**Fig 7:**
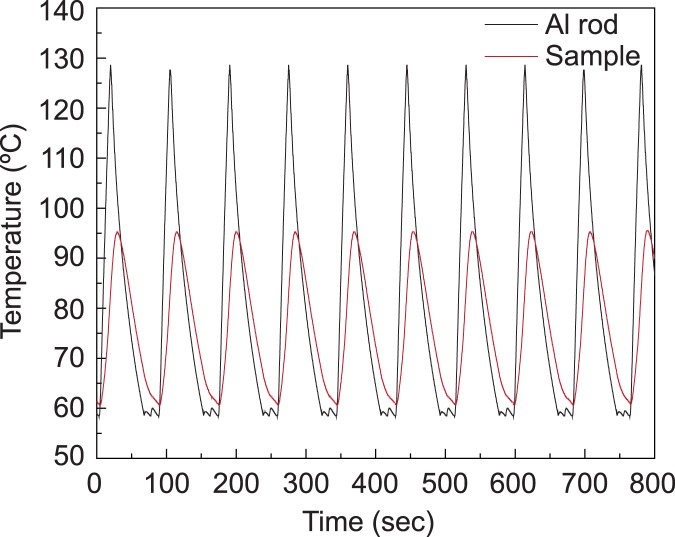
The temperature plot of the sample and the heating element for calibration. The plot is used to determine proper thermocycling control thresholds. The gray curve shows the temperature of the aluminum heating block; the red curve shows the temperature inside of the 20ul sample inside of the PCR tube.

Since the temperature of the sample cannot be taken directly from the inside of the PCR tube, the temperature must be measured at the heating element. Thus, a calibration of sample temperature is necessary and must be performed in the ambient environment where the PCR reaction will take place. A test setup is made with an additional temperature probe placed inside of the PCR tube, along with 20 microliters of water coated with 20 microliters of mineral oil to simulate a sample being present in the tube. The high and low temperature thresholds used for the feedback control by the microcontroller, are then adjusted until the temperature of the sample cycled between 60°C and 95°C, which is ideal for the primer and DNA sequence used in this work. The resulted calibration used the low threshold of 59°C and the high threshold of 125°C, the sample temperature of 60°C and 95°C, respectively, as shown in Fig 7.

### Quantitative viral detection using 3D manufactured qPCR device

**Fig 8:**
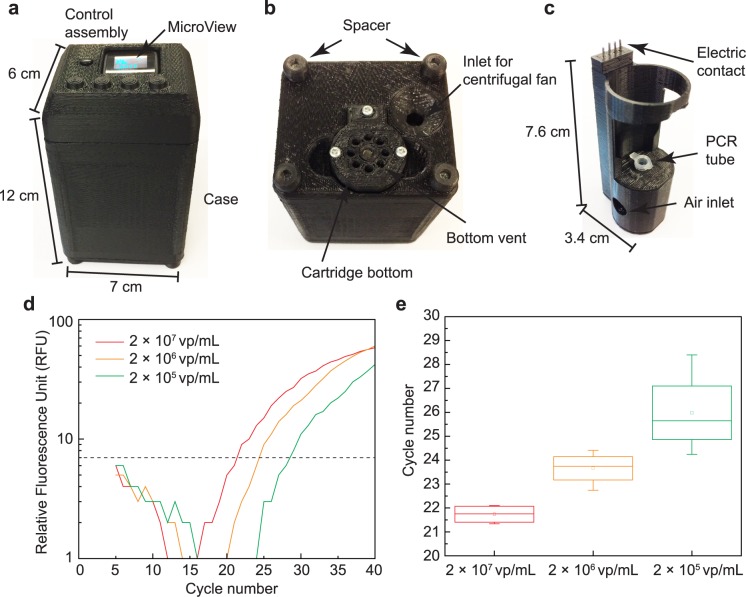
The photograph of 3D manufactured qPCR device and the resulted RT-qPCR from 3D manufactured qPCR device from three separate qPCR using 3 dilutions of target virus, 2 × 10^7^ vp/mL, 2 × 10^6^ vp/mL, and 2 × 10^5^ vp/mL. (a) The dimension of the qPCR device is 12 × 7 × 6 cm^3^. The MicroView shows the status of amplification cycle and fluorescence reading. (b) The bottom view of the qPCR device showing cartridge bottom and an air inlet for centrifugal fan. (c) The size of the cartridge that holds PCR tube during qPCR is 7.6 × 3.4 × 2.9 cm^3^. (d) Measured fluorescence readings show the shift in the intensity measurements corresponding to the differing concentrations of target virus. The threshold for determining C_q_ is also shown is a dotted line. (e) Measured Cq for three concentrations of target DNA. The mean and standard deviation of C_q_ are 21.74 ± 0.39, 23.66 ± 0.70, and 25.98 ± 1.75, for 2 × 10^7^ vp/mL, 2 × 10^6^ vp/mL, and 2 × 10^5^ vp/mL, respectively.

The overall dimensions of the final assembled device are approximately 12 × 7 × 6 cm^3^ with a mass of 214 g (Fig 8A). The bottom of the device has several critical elements (Fig 8B), including the opening which allows insertion of the cartridge, the air inlet for the centrifugal fan, and four spacers. These spacers allow for air to flow freely under the device when sitting on a flat surface, such as a benchtop. The dimensions of the cartridge are approximately 7.6 × 3.4 × 2.9 cm^3^ (Fig 8C). The PCR tube containing the sample is inserted into the cartridge allowing for contact with the heating block. During the PCR cooling phase, the ambient air is pulled into the bottom assembly through the inlet of the centrifugal fan (Fig 8B) and the fan blade accelerates the airflow toward the air inlet of the cartridge (Fig 8C). This increases the convection coefficient on the heating element to accelerate the cooling. Then, the air is pushed out from the cartridge through the bottom vent (Fig 8B). This airflow continues until the desired temperature is met for annealing and extension of DNA. The electrical wiring for the NiCr heating coil and thermistor run along the rear portion of the cartridge up to the electrical contacts located at the top of the cartridge (Fig 5B). These contacts mate with a connector located on the main board in the control assembly at the top of the device.

The main objective of quantitative real-time PCR is to determine the concentration of target virus present in the sample (Fig 8D and 8E). Three different concentrations of virus sample are prepared, 2 × 10^7^ vp/mL, 2 × 10^6^ vp/mL, and 2 × 10^5^ vp/mL. Experiments on each concentration are repeated 4 times with freshly prepared samples to verify the repeatability of the device. In order to determine the C_q_, MATLAB is used to automate the analysis. The intensity readings are sent from the device’s microcontroller to a PC using a serial connection and logged into MATLAB with the corresponding cycle number. The first step in the analysis is to omit the first 5 cycles to avoid reaction stabilizing artifacts. Following this, the set of data from multiple sets is normalized so that differences in baseline fluorescence are removed. A plot of three normalized curves is shown in Fig 8D. Each curve is then fit to a polynomial curve to smooth out the discrete steps caused by the digitization of the intensity measurements as well as to reduce noise on the measurements. The polynomials are then solved to find the corresponding cycle number where each curve crossed a threshold, or the C_q_. The results from three concentrations are shown in Fig 8E. The threshold is determined based on the noise level of the fluorescence baseline. The raw data from the photodiode, including the result from the negative control, is shown in S3 Fig. The negative control had no fluorescence increase as expected. The qPCR typically required 90 minutes to complete the entire process, from reverse-transcription to 40 cycles of PCR. The measured C_q_ (mean ± standard deviation) are 21.74 ± 0.39, 23.66 ± 0.70, and 25.98 ± 1.75, for 2 × 10^7^ vp/mL, 2 × 10^6^ vp/mL, and 2 × 10^5^ vp/mL, respectively. For each concentration the C_q_ clearly shifts between 2 and 3 cycles as one would expect for a ×10 dilution. However, the standard deviation in samples with 2 × 10^6^ vp/mL and 2 × 10^5^ vp/mL are significantly larger than that of 2 × 10^7^ vp/mL. This can be interpreted as a simple human error in accurate dilution. If the deviation is originating from the device, the original sample with 2 × 10^7^ vp/mL, supplied by a vendor, should have been detected with a large deviation as well, which is untrue according the data (standard deviation = 0.39 cycle). This can also explain the low cycle count (2–3 cycles) separation between C_q_ of ×10 dilutions. Ideally, the separation should be close to 3.32.

**Fig 9:**
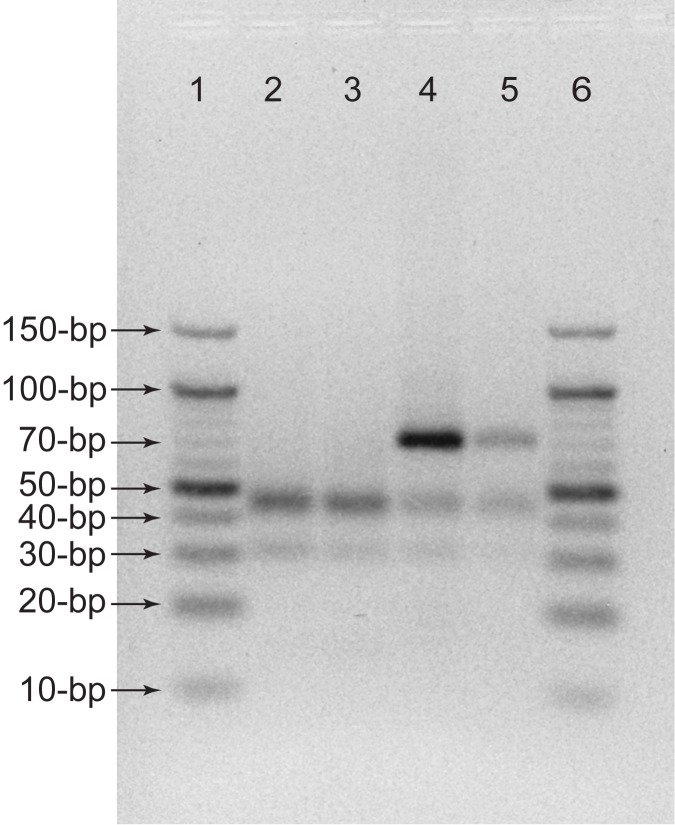
Comparison of conventional qPCR machine and 3D printed device using gel electrophoresis. Each lane included: (lane 1 and lane 6) 10-bp DNA ladders, (lane 2) negative control from conventional device, (lane 3) negative control from 3D printed device, (lane 4) 2 × 10^7^ vp/mL sample from conventional device, and (lane 5) 2 × 10^7^ vp/mL sample from 3D printed device.

The amplification using the presented qPCR device is confirmed by performing a gel electrophoresis. In Fig 9, the amplifications are compared with a conventional qPCR machine (Applied Biosystems 7900HT). A negative control and a sample with 2 × 10^7^ vp/mL are thermally cycled and read using both the conventional machine and the presented qPCR device. The negative control and virus-containing sample results from the 3D printed device matches those from the conventional machine as shown in Fig 9. The length of amplicon amplified used for this experiment is 68-bp and the band shown in two negative controls are primers, consistent with other RT-PCR performed on TaqMan samples in other literature [25].

## Conclusions and discussions

The presented device has met the goals for easy manufacture and quantitative viral detection. The custom parts, including circuit boards, mechanical housing and holders, and the aluminum heating block, are easily made by a 3D printer, a 3D CNC mill, and by hand using simple tools. The majority of electronic parts are simple off-the-shelf parts that can be easily purchased. The total estimated cost of a unit is ~300 USD. A breakdown of this cost is shown in supplemental data in S1 Table. The use of the 3D printer has allowed for rather complex components to be made in a rapid and low-cost fashion which greatly opens access to this qPCR device, as well as future medical devices. For example, the resulting system contains thirteen unique 3D designed and printed components, each of which would be out of reach for someone without access to a machine shop. The low standard deviation of C_q_ for 2 × 10^7^ vp/mL indicates that the quantification is highly repeatable in this device. The recorded C_q_ can be used as a calibration curve to quantify a patient sample with unknown amount of pathogen. The shift of C_q_ toward high cycle counts is observed for lower level concentrations, which indicates a successful quantification in the diluted sample as well.

Several challenges were addressed for the success of this project. One is the importance of the light path design. Our experimentation has shown that the layout of the light path is critical in obtaining an accurate fluorescent measurement. Also, the reasonable heating and cooling duration was a topic that needed to be investigated. In order to be consistent with the theme of this work, a simple fan-based cooling method is used, rather than adapting more elegant cooling methods, such as a Peltier device. However, the fluorescence-based quantitative reading requires a complete enclosure around the heating element and the sample to reduce background noise in the photodiode reading. To satisfy both conditions, a centrifugal fan design is adapted to redirect the air inlet to the air vent, which inhibits any directional external light exposure to the photodiode through the air inlet and vent. As a part of future work, new heating and cooling mechanisms are being investigated to reduce the total qPCR duration. One important topic that is not addressed in the work is sample preparation. In order for a qPCR diagnostic device to be highly relevant in a resource-limited setting, it is essential to have an easy and low-cost solution for sample preparation, prior to qPCR detection. This is particularly true when using a blood sample that can inhibit PCR. The DNA or RNA needs to be extracted from the raw sample before loading the sample into the qPCR device. We are working toward integrating a simple sample preparations scheme to enhance the usefulness of the presented work.

## Supporting information

S1 Fig The digital design files corresponding to each parts of 3D assembly. The 3D model is given in STL file format, a file type commonly used for rapid prototyping and 3D printing. The file format for circuit boards, Control Board and Main Board, is DXF, that is compatible with 3D CNC mill.
(EPS)

S2 Fig The circuitry and circuit board used in the control assembly. (a) The control board consists of switches and pull-up resistors, that offers user interface with the microcontroller for initiation and control of qPCR. (b) The main board consists of a microcontroller, an amplifier for photodiode, a voltage regulator, and two relays for motor and heater controller. The circuit board layout (c) for control board, and (d) for main board are used in a 3D CNC mill for circuit board fabrication.
(EPS)

S3 Fig Measured qPCR fluorescence readings including the negative control. The raw data from the photodiode is read in voltage and is plotted after baseline subtraction. This data is used to produce the log-scale qPCR plot in Fig 8.
(EPS)

S1 File This file contains all the 3D models in STL format. The included files are: Bottom Plate.stl, Button Piece.stl, Cartridge Housing.stl, Case.stl, Emission Filter Holder.stl, Faceplate.stl, Fan Blade.stl, Heater Holder.stl, Motor Holder.stl, Photodiode Assembly Mount.stl, Photodiode Holder.stl, Spacer.stl, and Wire Cover.stl. The assembly order can be found in S1 Fig.
(ZIP)

S2 File The file contains DIPTRACE file for control board and main board. Also, DXF files are provided for 3D CNC milling.
(ZIP)

S3 File The Arduino code is for programming the microcontroller for qPCR. (ZIP)

S4 File This file contains all the source CAD files which includes those to create the STL files for printing. Also, the 3D assembly of the device (3D Printed PCR.iam) as well as subassemblies and individual parts are contained in this file.
(ZIP)

S1 Table The list of components used in the qPCR device. (EPS)
