# Supplementary figures and images for: 3D printing and milling a real-time PCR device for infectious disease diagnostics
Source: PLoS One. 2017 Jun 6;12(6):e0179133. doi: 10.1371/journal.pone.0179133 (PMC5460903; doi:10.1371/journal.pone.0179133)

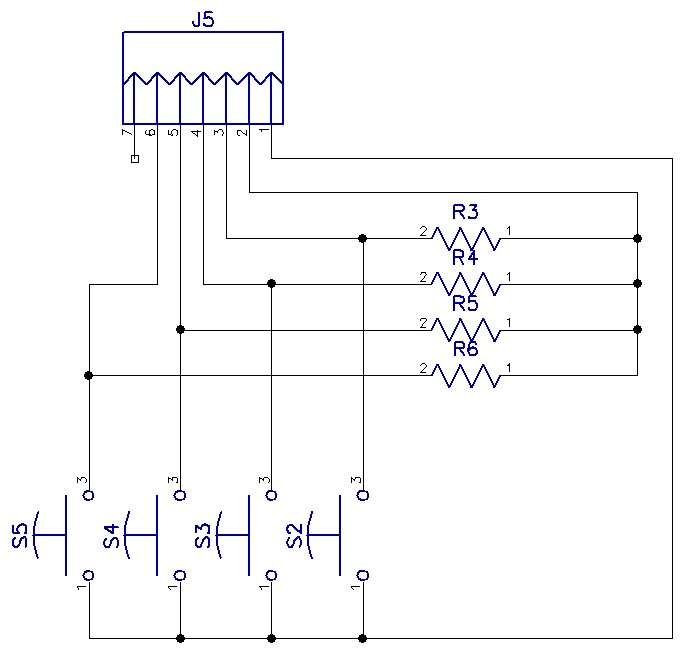

Supplement: S2 File — Also, DXF files are provided for 3D CNC milling. (ZIP) [file pone.0179133.s005.zip › Circuit Board/Control Board Schematic.jpg]

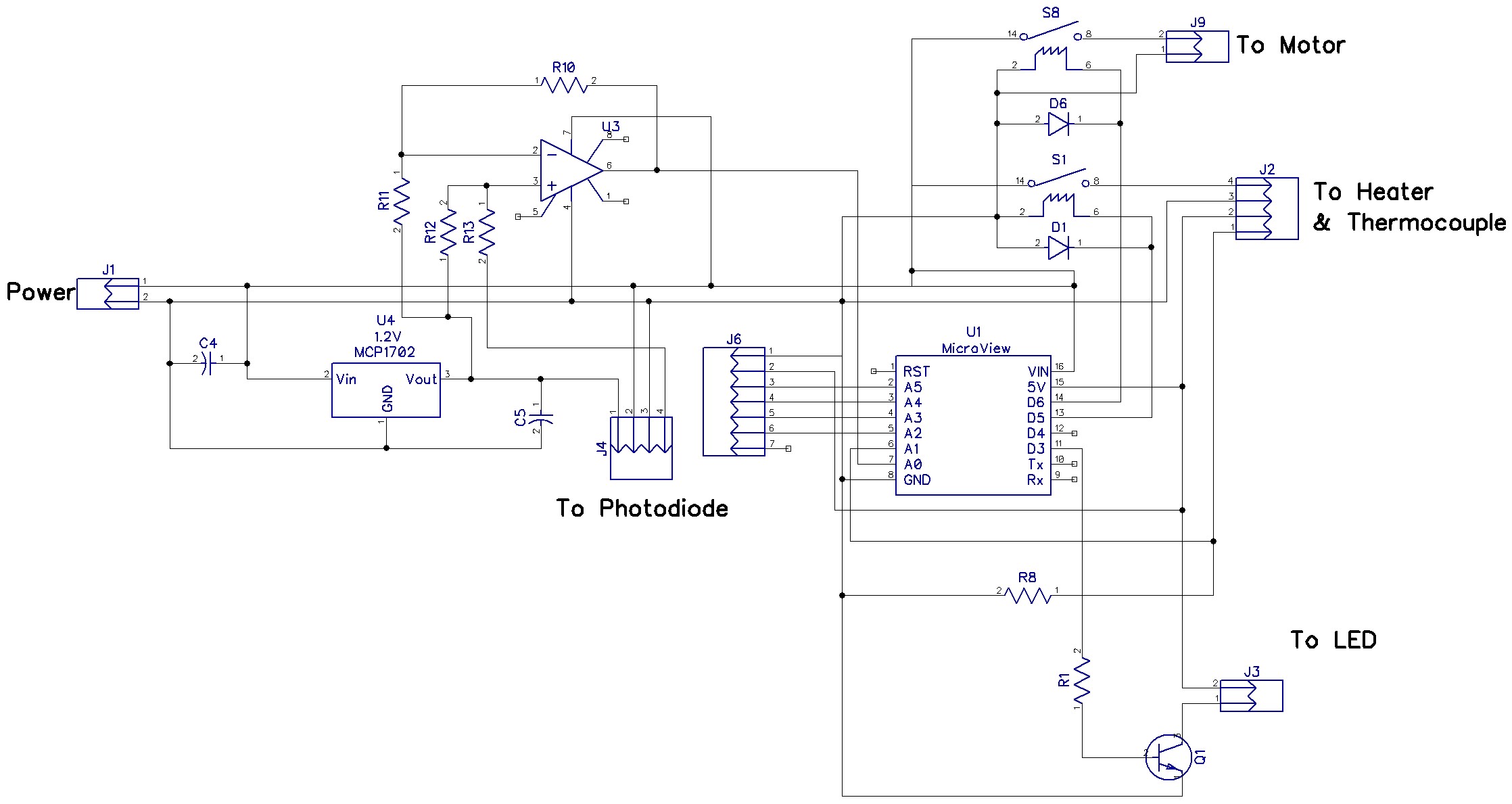

Supplement: S2 File — Also, DXF files are provided for 3D CNC milling. (ZIP) [file pone.0179133.s005.zip › Circuit Board/Main Board Schematic.jpg]
